# Regulation of Na^+^/H^+^ exchangers, Na^+^/K^+^ transporters, and lignin biosynthesis genes, along with lignin accumulation, sodium extrusion, and antioxidant defense, confers salt tolerance in alfalfa

**DOI:** 10.3389/fpls.2022.1041764

**Published:** 2022-11-07

**Authors:** Md Atikur Rahman, Jae Hoon Woo, Sang-Hoon Lee, Hyung Soo Park, Ahmad Humayan Kabir, Ali Raza, Ayman El Sabagh, Ki-Won Lee

**Affiliations:** ^1^ Grassland and Forage Division, National Institute of Animal Science, Rural Development Administration, Cheonan, South Korea; ^2^ Molecular Plant Physiology Laboratory, Department of Botany, University of Rajshahi, Rajshahi, Bangladesh; ^3^ Department of Genetics, University of Georgia, Athens, GA, United States; ^4^ College of Agriculture, Oil Crops Research Institute, Fujian Agriculture and Forestry University (FAFU), Fuzhou, China; ^5^ Faculty of Agriculture, Department of Field Crops, Siirt University, Siirt, Turkey; ^6^ Department of Agronomy, Faculty of Agriculture, Kafrelsheikh University, Kafr El-Shaikh, Egypt

**Keywords:** abiotic stress, antioxidant defense, ion exchanger, salinity stress, sodium transporter, phenylpropanoid

## Abstract

Accumulation of high sodium (Na^+^) leads to disruption of metabolic processes and decline in plant growth and productivity. Therefore, this study was undertaken to clarify how Na^+^/H^+^ exchangers and Na^+^/K^+^ transporter genes contribute to Na^+^ homeostasis and the substantial involvement of lignin biosynthesis genes in salt tolerance in alfalfa (*Medicago sativa* L.), which is poorly understood. In this study, high Na^+^ exhibited a substantial reduction of morphophysiological indices and induced oxidative stress indicators in Xingjiang Daye (XJD; sensitive genotype), while Zhongmu (ZM; tolerant genotype) remained unaffected. The higher accumulation of Na^+^ and the lower accumulation of K^+^ and K^+^/(Na^+^ + K^+^) ratio were found in roots and shoots of XJD compared with ZM under salt stress. The ZM genotype showed a high expression of *SOS1* (*salt overly sensitive 1*), *NHX1* (*sodium/hydrogen exchanger 1*), and *HKT1* (*high-affinity potassium transporter 1*), which were involved in K^+^ accumulation and excess Na^+^ extrusion from the cells compared with XJD. The lignin accumulation was higher in the salt-adapted ZM genotype than the sensitive XJD genotype. Consequently, several lignin biosynthesis–related genes including *4CL2, CCoAOMT, COMT, CCR, C4H*, *PAL1*, and *PRX1* exhibited higher mRNA expression in salt-tolerant ZM compared with XJD. Moreover, antioxidant enzyme (catalase, superoxide dismutase, ascorbate peroxidase, and glutathione reductase) activity was higher in ZM relative to XJD. This result suggests that high antioxidant provided the defense against oxidative damages in ZM, whereas low enzyme activity with high Na^+^ triggered the oxidative damage in XJD. These findings together illustrate the ion exchanger, antiporter, and lignin biosysthetic genes involving mechanistic insights into differential salt tolerance in alfalfa.

## Introduction

Increasing salts (NaCl, NaSO_4_ Na_2_CO_3_, and NaHCO_3_) in agricultural soils because of salinization and/or alkalization is a serious global issue for plant growth and productivity (Fang et al., 2021; Ma et al., 2022; Raza et al., 2022b). Salt stress induces osmotic imbalance and Na^+^ toxicity–mediated oxidative stress, which negatively affects plant physiochemical responses, photosystem II (PSII), and chlorophyll a fluorescence (Khan et al., 2022; Khatri and Rathore, 2022). Protecting from the adverse effects and managing the osmotic stress, a plant accumulates several compatible solutes (proline, soluble sugar, and glycine betaine) known as osmoprotectants (Ghosh et al., 2021; Chauhan et al., 2022). In addition, antioxidant enzymes, namely, superoxide dismutase (SOD), catalase (CAT), ascorbate peroxidase (APX), and glutathione reductase (GR), protect plants from cellular injury and lipid peroxidation through scavenging of reactive oxygen species (ROS) under salt stress conditions (Carrasco-Ríos and Pinto, 2014). The Na^+^ is a critical player in salt stress–related studies in plants. Excess Na^+^ accumulation in the cytosol can also be toxic for plants. The transport of Na^+^ and potassium ions (K^+^) across the plasma membrane is regulated by a membrane-associated transportation system involving H^+^-ATPase, K^+^ channels, Na^+^/K^+^ transporters, and a Na^+^/H^+^ antiporter (Almeida et al., 2017). The influx of Na^+^ in the root system depolarizes the plasma membrane, which disturbs K^+^ influx, causing K^+^ loss and a K^+^-dependent enzymatic defense system in plants (Khan et al., 2021).

To cope with the excess accumulation of Na^+^ with toxic effect, salt-tolerant genotypes exhibit a high tolerance to salt stress by maintaining K^+^/Na^+^ ratio in the cytosol compared with salt-susceptible genotypes (Almeida et al., 2017). Though responses to salt tolerance vary from genotype to genotype, in most cases, plants rely on the regulation of Na^+^ and K^+^ transporters and H^+^ pumps, which generate the driving force for the Na^+^ and K^+^ transport. The three key transporters, including *salt overly sensitive* (*SOS*), *Na^+^/H^+^ exchanger* (*NHX*), and *high-affinity potassium transporter* (*HKT*), are involved in the transport, translocation, and compartmentation of ions (Na^+^ and K^+^) into cells and/or organelles (Fan et al., 2015). The SOS pathway is recognized for salt stress signaling and tolerance plant system (Almeida et al., 2017). *SOS1* is a plasma membrane Na^+^/H^+^ antiporter that regulates Na^+^ extrusion from the cytoplasm and mediates long-distance (roots–shoots) Na^+^ transport (Xie et al., 2022). *SOS2*, a serine/threonine protein kinase, activates the response of the plasma membrane Na^+^/H^+^ antiporter *SOS1* through phosphorylation (Almeida et al., 2017). *SOS3* is a calcium sensor protein induced after salt stress–involving Ca^2+^ signal and enhances the expression of *SOS1* through the interaction of the *SOS2–SOS3* kinase complex that leads to efflux of Na^+^ from the cells (Xie et al., 2022).

The SOS signaling pathway–related genes *SOS1, SOS2/CIPK24*, and *SOS3* have been characterized in *Arabidopsis thaliana* (Yin et al., 2019; Chai et al., 2020), *Oryza sativa* (Zhou et al., 2013), and halophyte (*Salicornia brachiata*) (Goyal et al., 2013). Moreover, it has been reported that *AtSOS1* enhances salt stress tolerance in Arabidopsis, whereas the *sos1* mutant exhibited a significant decrease in salt tolerance (Oh et al., 2010). Likewise, a relevant study also found in rice the rss2 mutant (Zhou et al., 2013). However, among the SOSs, *SOS1* is the key player that controls salt tolerance. Another Na^+^/H^+^ antiporter, the NHX gene family, is involved in Na^+^ defense mechanism through vacuoles where the H^+^ gradient is utilized as a driving force (Fu et al., 2020). Besides Na^+^ transporter genes, the role of K^+^ transporter genes in salt stress studies is also important. The HKT genes belong to the potassium transport family; *HKT1* deals with salt stress through regulation of Na^+^ and K^+^ homeostasis at a cellular and molecular levels (Ali et al., 2019). Recently, *TmHKT1;5* was found to be involved in high-affinity K^+^‐dependent Na^+^ transport processes, and major shoot Na^+^ is prohibited while external K^+^ is in excess compared with Na^+^ (Byrt et al., 2007). Despite these recent advancements, the deep insights on ion transporter genes–mediated salt tolerance in forage legume species are yet to be disclosed.

Lignin is crucial for cell wall composition, structural support, and stress adaptation in plants (Liu et al., 2018). Molecular mechanisms involving lignin accumulation and salt adaptation is well documented in the model plant *Arabidopsis* (Chun et al., 2019). Transcriptional level of candidate genes linked to root lignification, cell wall solidification, and thickening of vascular tissues changes under salinity (Byrt et al., 2018). RNA-seq analysis in bermudagrass genotypes differing in salt tolerance identified the candidate genes involved in alterations of lignin synthesis and ROS homeostasis controlled by peroxidases (Hu et al., 2015). The cell wall lignin deposition in endodermal and exodermal cells changes under salt stress (Byrt et al., 2018). These molecular and physiological changes enhance adaptation in plants to salt stress by preventing water loss and altering ion (i.e., Na^+^, Cl^-^, and K^+^) transporting pathways. Furthermore, the regulation of ion transporters under salt stress alters the cell wall–ion binding capability and enzymetic responses (Huang et al., 2020).

For example, the binding of Na^+^ to cell wall components influences the transport of Na^+^ and other ions, affects cell wall–modifying enzymes, and increases the deposition of lignin and suberin in endodermal–exodermal cells (Byrt et al., 2018). These ion transporters and their regulatory mechanisms are involved in salt tolerance in plants. Phenylpropanoid pathway–related enzymes catalyze multiple reactions, and some are involved in lignin biosynthesis. For instance, phenylalanine ammonia-lyase (PAL), cinnamate 4-hydroxylase (C4H), 4-coumarate CoA ligase (4CL), cinnamoyl CoA reductase (CCR), caffeoyl CoA O-methyltransferase (CCoAOMT), ferulate 5-hydroxylase (F5H), caffeate 3-O-methyltransferase (COMT), and cinnamyl alcohol dehydrogenase (CAD) are involved in monolignol synthesis and salt adaptation in *Arabidopsis* (Chun et al., 2019). The induction of lignification may vary in response to salinity in different plant species. However, the role of lignin biosynthetic genes in salt stress tolerance in legume crops is not fully understood.

Alfalfa (*Medicago sativa* L.) is a perennial forage legume, widely cultivated as an animal fodder because of high-protein content and biomass production (Coburn et al., 2021). Alfalfa provides beneficial impacts on sustainable agriculture, adds N_2_ to the soils, increases organic matter, and decreases the reliance on N_2_ fertilizers (Song et al., 2021). Alfalfa shows superior tolerance to abiotic stresses, including drought, heat, and oxidative stress (Matthews et al., 2019; Ma et al., 2021). Therefore, it might be used as a promising source of germplasms for molecular breeding with enhanced tolerance to multiple abiotic stresses. However, the investigation of potential responses of ion exchangers, transporter/antiporters, and lignin genes with the differential salt tolerance might be useful to the discovery of molecular mechanisms underlying the greater salt tolerance in alfalfa and other legume species. Therefore, this study was focused on understanding the molecular mechanisms that enhanced salt tolerance in alfalfa and discovering the potential strategies to develop or breed salt-resilient alfalfa plants.

## Material and methods

### Plant cultivation and salt treatment

The sterilized seeds of two alfalfa (*M. sativa* L.) genotypes having differential tolerance to salt stress, namely, Zhongmu (ZM; salt tolerant, plant height 35–40 cm, leaf round trifoliate) and Xingjiang Daye (XJD; salt sensitive, plant height 30–35 cm, leaf slender trifoliate), were germinated in a plastic tray. The 3-day-old alfalfa seedling was transferred to a half-strength Hoagland nutrient solution (Hoagland and Arnold, 1950). Salt concentration of 150 mM was selected after trial experiments. The 150-mM sodium chloride (NaCl) was added to the nutrient solution. The treatments were considered as ZM 0 (control), ZM 150 mM NaCl, XJD 0 (control), and XJD 150 mM NaCl. In all treatments, the pH was adjusted at 6.0. The alfalfa seedlings were cultivated individually, with three biological repetitions in plastic pots (1.0 L). A total of nine seedlings were placed in each pot, with a completely randomized block design. The nutrient solution was replaced every 2 days, and the treatments were maintained for 7 days prior to data collection. The alfalfa seedlings were cultivated in a control growth chamber with an optimum temperature at 25°C, white fluorescent light (480 μmol m^−2^ s^−1^) with a 14-h photoperiod, and 60%–65% relative humidity.

### Measurement of physiological attributes

The leaf chlorophyll concentration was measured using a SPAD-502 meter (Minolta, Japan). The maximum yield of PSII (Fv/Fm) was determined using a portable fluorometer (PAM 2500, Effeltrich, Germany). The plants were adapted at dark for 30 min prior to data collection.

The leaf fresh weight (FW), turgid weight (TW), and dry weight (DW) were measured using an electronic balance (Mettler PM 200, Switzerland), and relative water content (RWC%) was calculated using the formula as follows:


RWC%=(FW−DW)/(TW−DW)×100

### Localization of superoxide radicle and hydrogen peroxide using fluorescent histochemical staining

Superoxide radical ( ${\text{O}}_{2}^{•-}$ ) and hydrogen peroxide (H_2_O_2_) were localized following the method (Sandalio et al., 2008). Alfalfa root tissue was stained using a specific probe dihydroethidium (DHE; Sigma-Aldrich). Alfalfa root tips were incubated at 37°C with Tris-HCl (10 mM, pH 7.4) containing 10 μM DHE in the dark for 30 min and washed thrice with fresh buffer. The fluorescence of the ${\text{O}}_{2}^{•-}$ -specific probe DHE was visualized in a florescent microscope (CLS-01-00076, Logos Biosystem, Inc., South Korea) by excitation and emission at 488 and 520 nm, respectively. Fluorescence intensity of H_2_O_2_ was visualized using H_2_O_2_ detection probe 2,7-dichlorofluorescein diacetate (DCF-DA, Sigma-Aldrich). Briefly, 50 μM DCF-DA was used for incubating alfalfa roots; the same incubation environment and time duration of ${\text{O}}_{2}^{•-}$ were followed for H_2_O_2_ as well. Root tips were washed three times with deionized water, and excess water was removed by tissue paper (Kimteck, South Korea). The reaction of H_2_O_2_ in the presence of florescent DCF-DA molecules was monitored using a fluorescence microscope (CLS-01-00076, Logos Biosystem, Inc., South Korea) by excitation at 480 nm and emission at 530 nm.

### Determination of elemental concentration

The harvested roots were washed properly using Milli-Q water to remove excess salt from the root surface. The root and shoot samples were dried at 80°C for 72 h, individually weighted, and digested using a solution (HClO_4_/HNO_3,_ 1/3, v/v) as described previously (Haque et al., 2021). The soluble ion (Na^+^ and K^+^) concentrations were determined using inductively coupled plasma mass spectrometry (ICP-MS; Optima 5300DU, Perkin Elmer, USA). Three individual replications of root and shoot samples were considered for the ICP-MS analysis.

### Quantification of lignin content

A cysteine-assisted sulfuric acid method was followed to measure lignin content in plant biomass (Lu et al., 2021). Briefly, 80–100 mg of grinded plant sample was taken in a glass beaker. Then, 1 ml 72% sulfuric acid containing 10% L-cysteine (1 g Cys in 10 ml 72% H_2_SO_4_) was added. The glass beaker was sealed with fresh polythene and rubber band, kept it on a rotary shaker (50 rpm) at 24°C for 1 h and mixed well. The solution mixture was diluted with DEPC-treated water to a volume of 5–10 ml in a glass beaker. The absorbance of solution was read at 283 nm (A283) using UV-Spectra Max i3x (Minimax 300, San Jose, USA).

### Determination of proline and soluble sugar content

Proline accumulation was measured following the methods described previously (Bates et al., 1973; Rahman et al., 2014). Briefly, 100 mg of plant tissue was homogenized in 5 ml of 3% sulfosalicylic acid. The homogenate was centrifuged at 10,000 rpm for 15 min. Supernatant of the solution, acid ninhydrine, and glacial acid were mixed 1:1:1 (v/v) in a new tube and incubated in water bath for 1 h then kept the mixture on ice for 5 min. Toluene was added to extract the reaction mixture, and the absorbance was read at 520 nm. Proline content was calculated using L-proline as a standard. Soluble sugar was quantified using the anthrone reagent (Merck, Germany). For this, 100 mg of plant tissue was mixed with 5 ml of 100% acetone and centrifuged for 20 min. The acetone-extracted interfering pigment was discarded, and the precipitate was recovered using 80% ethanol with centrifugation. Supernatant was mixed with 72% H_2_SO_4_ acid containing ice-cold anthrone (1:5, v/v). The mixture was boiled at 100°C water bath for 15 min and then kept on ice for 2 min. The absorbance of the solution mixture was checked at 630 nm compared with the sugar blank sample. Soluble sugar was calculated following D-glucose as normal.

### Analysis of antioxidant enzymes

Antioxidant enzyme activities were quantified in the root tissue following the protocols (Rahman et al., 2022). Briefly, 100 mg of pulverized plant sample was homogenized with 50 mM potassium phosphate (KP) buffer (pH 7.0) and 5 mM β-mercaptoethanol (β-ME). The homogenate was centrifuged at 12,500 ×*g* for 20 min; the supernatant was collected and used as enzyme extract. Per unit of SOD (EC 1.15.1.1) was estimated by adding of extract (100 μl) to 1 ml of EDTA (0.1 mM), NaHCO_3_ (50 mM, pH 9.8), and epinephrine (0.6 mM). The formation of adreno-chrome from the enzymatic reaction was read at 475 nm. CAT (EC: 1.11.1.6) activity was determined using the KP buffer containing hydrogen peroxide (6%). Decrease of absorbance was monitored at 240 nm for 1 min. The CAT activity was calculated using an extinction coefficient of 39.4 M^−1^ cm^−1^. APX (EC 1.11.1.11) was measure following the protocol (Lee et al., 2017). The enzyme activity was quantified by combining the extract (100 μl) with EDTA (0.1 mM), the KP buffer (50 mM, pH 7.0), hydrogen peroxide (0.1 mM), and ascorbic acid (0.5 mM). The absorbance of the solution mixture was measured at 290 mm, and the activity was calculated considering the extinction coefficient of 2.8 M^−1^ cm^−1^. The GR (EC 1.6.4.2) activity was checked by adding 1 ml of 0.2 M KP buffer (pH 7.0) that contained EDTA (1 mM), oxidized glutathione (GSSG, 20 mM), NADPH (0.2 mM), and enzyme extract. The absorbance of solution was read at 340 nm, and GR activity was calculated using an extinction coefficient of 6.12 M^−1^ cm^−1^.

### RNA extraction and gene expression analysis

Total RNA was extracted from the alfalfa sample using the RNA extraction kit (QIAGEN, Maryland, USA). Briefly, 100 mg of pulverized plant tissue was homogenized with the extraction buffer including 1% β-ME. Following washing steps, total RNA was recovered. RNA concentration was measured using UV/Vis spectrophotometer (UV Drop-99, Taipei, Taiwan), while the concentration ≥300 ng/μl was considered for further molecular analysis. RNA quality was checked by gel electrophoresis. The extracted RNA was used to synthesize the first strand of cDNA. The real-time PCR was performed using CFX-96™ real-time PCR (BIORAD) to check the expression of targeted *Medicago* genes using gene-specific primers (**Supplementary Table 1**). The targeted genes were amplified following PCR programs as follows: 95°C for 30 s, followed by 40 cycles at 95°C for 5 s, 60°C for 30 s, and extension at 60°C for 1 min. The housekeeping gene *MsActin* was considered as an internal control. Gene expression was calculated following the 2^−ΔΔCt^ method (Livak and Schmittgen, 2001).

### Statistical analysis

The physiological and gene expression data were subjected to ANOVA. The mean differences were measured by Tukey’s honestly significant difference test. Differences at p ≤ 0.05 were considered as significant. In addition, the graphical figures were constructed using the GraphPad Prism program (version 9.0). All the results were presented as mean value ± standard error of at least three biological replications.

## Results

### Effects of salt stress on morphological and physiological parameters

**Figure 1:**
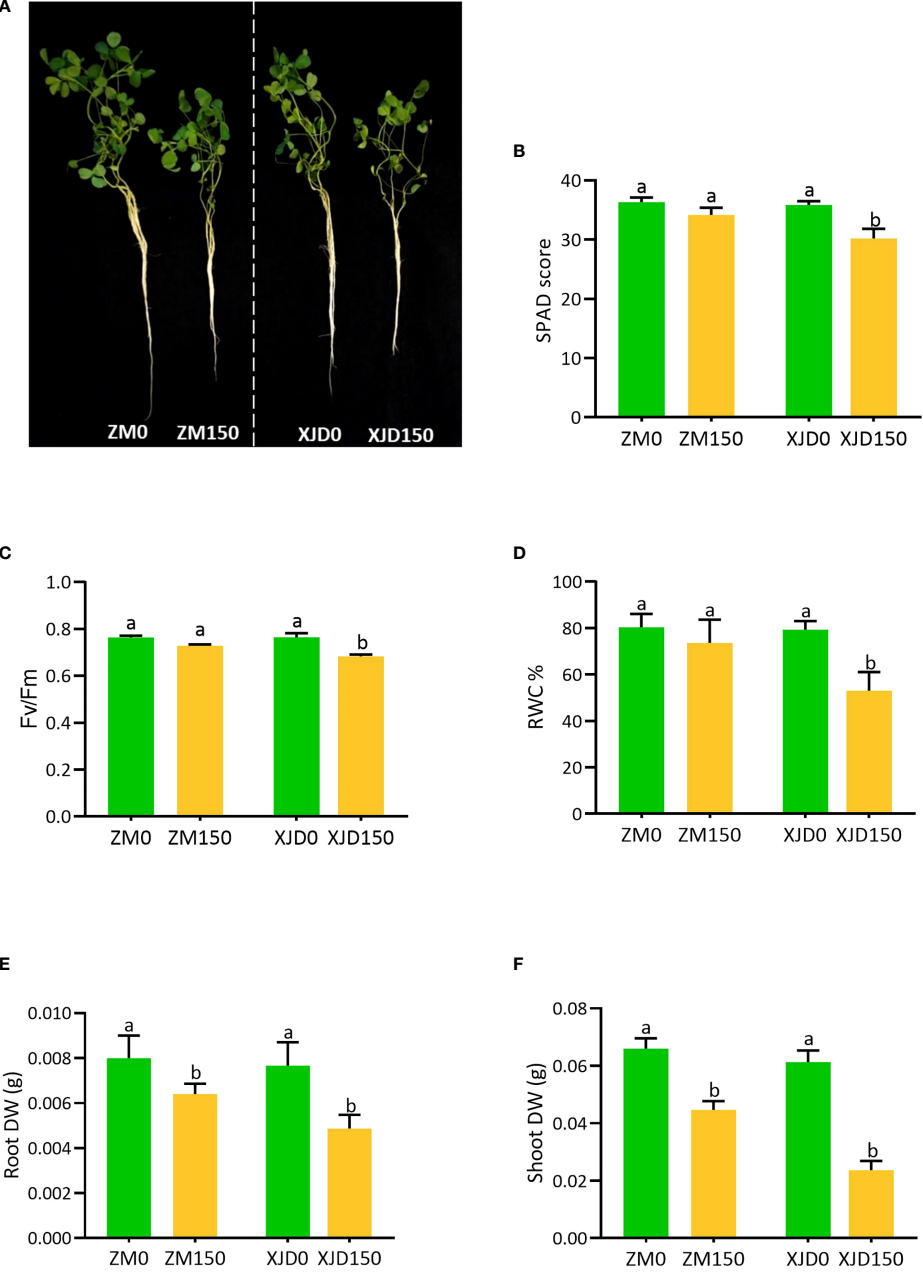
The variation of morphological and physiological indices in two contrasting alfalfa genotypes in response to salt stress. Different plant phenotypes **(A)**, SPAD score **(B)**, Fv/Fm **(C)**, RWC% **(D)**, Root DW **(E)**, and Shoot DW **(F)**, changes in Zhongmu (ZM) and Xingjiang Daye (XJD) under salt stress. The numeric zero (0) indicates salt-untreated (control) plant, while the numeric 150 indicates 150 mM salt treatment. Different letters above the column bar indicate significant differences among the means ± SD of treatments (n = 3). Data were analyzed by ANOVA and Tukey’s tests at p ≤ 0.05 significant level.

Salt stress caused a morphophysiological disturbance in alfalfa. A phenotypic difference was clearly observed between the two contrasting genotypes in response to salt stress (**Figure 1A**). The SPAD score, Fv/Fm ratio, and relative water content (RWC%) were significantly declined in leaves of XJD compared with salt-untreated (control) plants. In contrast, ZM showed no significant differences for these physiological parameters under salt stress (**Figures 1B-D**). The dry biomasses of root and shoot were significantly decreased in both genotypes (ZM and XJD) relative to control, while the salt effect was severe in XJD (**Figures 1E, F**).

### Alterations of reactive oxygen species generation under salt stress

**Figure 2:**
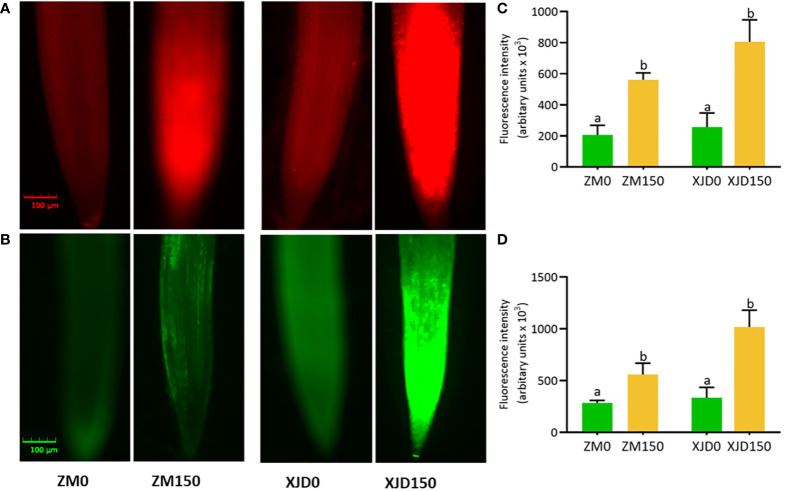
Accumulation of superoxide radical ( ${\text{O}}_{2}^{•-}$ ) and hydrogen peroxide (H_2_O_2_) in two contrasting alfalfa roots in response to salt stress. Visualization of fluorescence of ${\text{O}}_{2}^{•-}$ -specific probe dihydroethidium (DHE) **(A)** and H_2_O_2_-specific probe 2,7-dichlorofluorescein diacetate (DCF-DA) in root tips **(B)**. Fluorescence intensity of ${\text{O}}_{2}^{•-}$ **(C)**, and H_2_O_2_ **(D)**. The ${\text{O}}_{2}^{•-}$ and H_2_O_2_ are automatically measured by florescent microscope (CLS-01-00076, Logos Biosystem, Inc., South Korea). Pictures of stained roots were taken at 20× magnification. Scale bar = 100 μm. Different letters above the column bar indicate significant differences among the means ± SD of treatments (n = 3). Data were analyzed by ANOVA and Tukey’s tests at p ≤ 0.05 significant level.

The fluorescent histochemical staining demonstrated that ${\text{O}}_{2}^{•-}$ and H_2_O_2_ were markedly induced by salt stress in alfalfa root tips (**Figures 2A, B**). The fluorescence intensities of ROS ( ${\text{O}}_{2}^{•-}$ and H_2_O_2_) were significantly elevated by salt stress in alfalfa roots. The ${\text{O}}_{2}^{•-}$ and H_2_O_2_ intensities were prominently higher in XJD root tips compared with ZM, while untreated control plants showed no significant difference (**Figures 2C, D**).

### Accumulation of sodium (Na^+^) and potassium (K^+^) ions in the root and shoot

**Figure 3:**
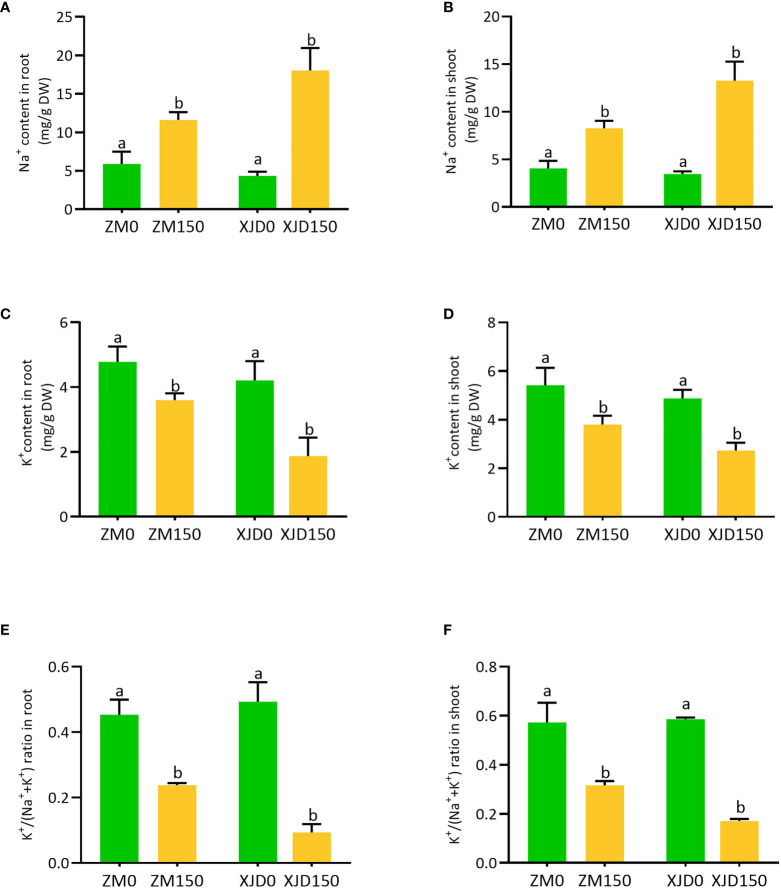
Elemental concentration in two contrasting alfalfa under salt stress. The Na^+^ in roots **(A)**, Na^+^ in shoots **(B)**, K^+^ in roots **(C)**, K^+^ in shoots **(D)**, K^+^/(Na^+^ + K^+^) ratio in roots **(E)**, and K^+^/(Na^+^ + K^+^) ratio in shoots **(F)**, in Zhongmu (ZM) and Xingjiang Daye (XJD) under salt stress. The numeric zero (0) indicates salt-untreated (control) plant, while the numeric 150 indicates 150 mM salt treatment. Different letters above the column bar indicate significant differences among the means ± SD of treatments (n = 3). Data were analyzed by ANOVA and Tukey’s tests at p ≤ 0.05 significant level.

The concentrations of Na^+^, K^+^, and K^+^/(Na^+^ + K^+^) ratio were drastically changed in two contrasting alfalfa genotypes following salt treatment (**Figure 3**). The Na^+^ accumulation was significantly higher in XJD root and shoot than ZM, while untreated control plants exhibited no considerable variation (**Figures 3A, B**). In contrast, K^+^ accumulations were substantially declined in the root and shoot of XJD compared with ZM (**Figures 3C, D**). As a consequence, the K^+^/(Na^+^ + K^+^) ratios were marked as lower in both the root and shoot of XJD than in the ZM genotype (**Figures 3E, F**).

### Lignin deposition in response to salt stress

**Figure 4:**
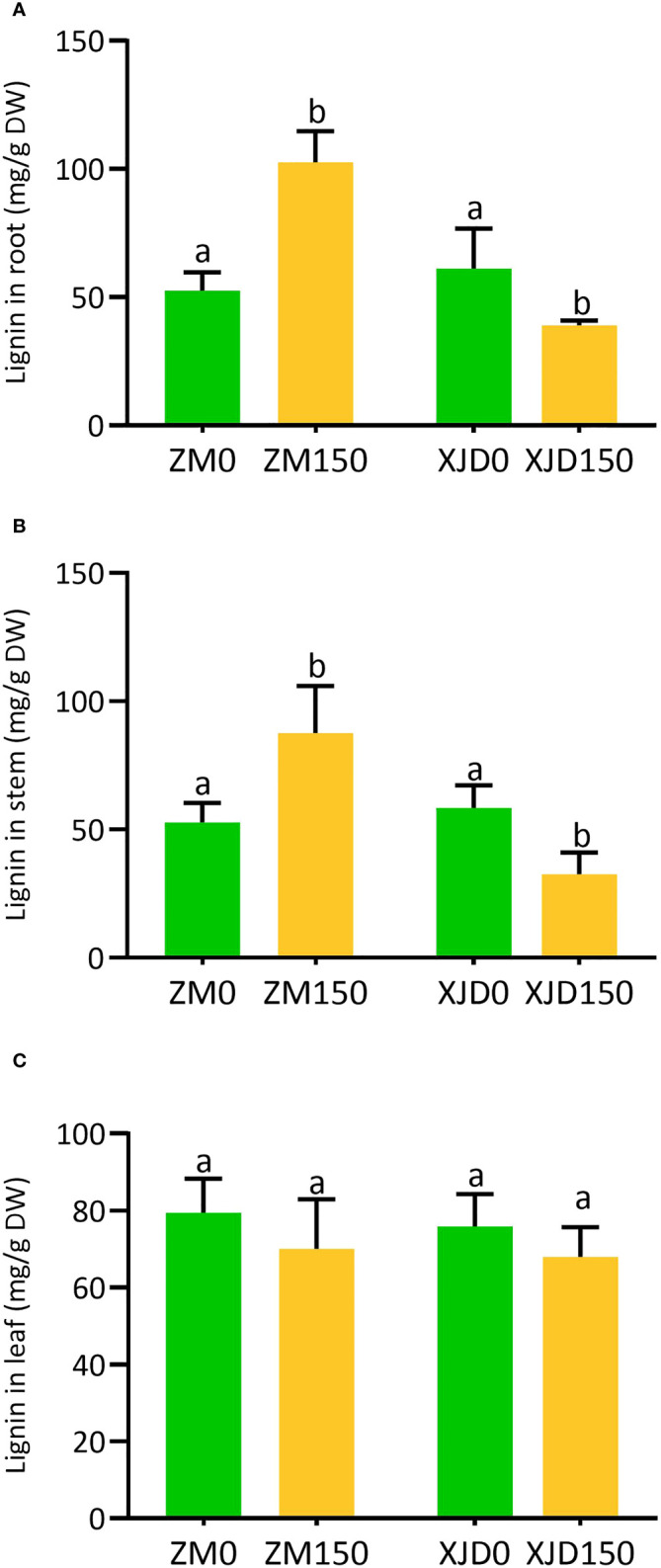
Lignin accumulation in two contrasting alfalfa under salt stress. Regulation of lignin in roots **(A)**, stems **(B)**, and leaves **(C)** of Zhongmu (ZM) and Xingjiang Daye (XJD) under salt stress. The numeric zero (0) indicates salt-untreated (control) plant, while the numeric 150 indicates 150 mM salt treatment. Different letters above the column bar indicate significant differences among the means ± SD of treatments (n = 3). Data were analyzed by ANOVA and Tukey’s tests at p ≤ 0.05 significant level.

Salt stress considerably regulated lignin deposition in the root, stem, and leaf of two contrasting alfalfa genotypes (**Figure 4**). The lignin deposition efficiency under salt stress was significantly declined in the root of XJD compared with normal conditions (**Figure 4A**). In contrast, the lignin deposition significantly increased in ZM compared with XJD in response to salt (**Figure 4B**). The accumulation of lignin was also higher in the stem of ZM, while it declined in XJD under salt stress (**Figure 4B**). However, lignin contents were not significantly different in the leaf between the two genotypes and/or among the treatments (**Figure 4C**).

### Expression of sodium and potassium transporter genes under salt stress

**Figure 5:**
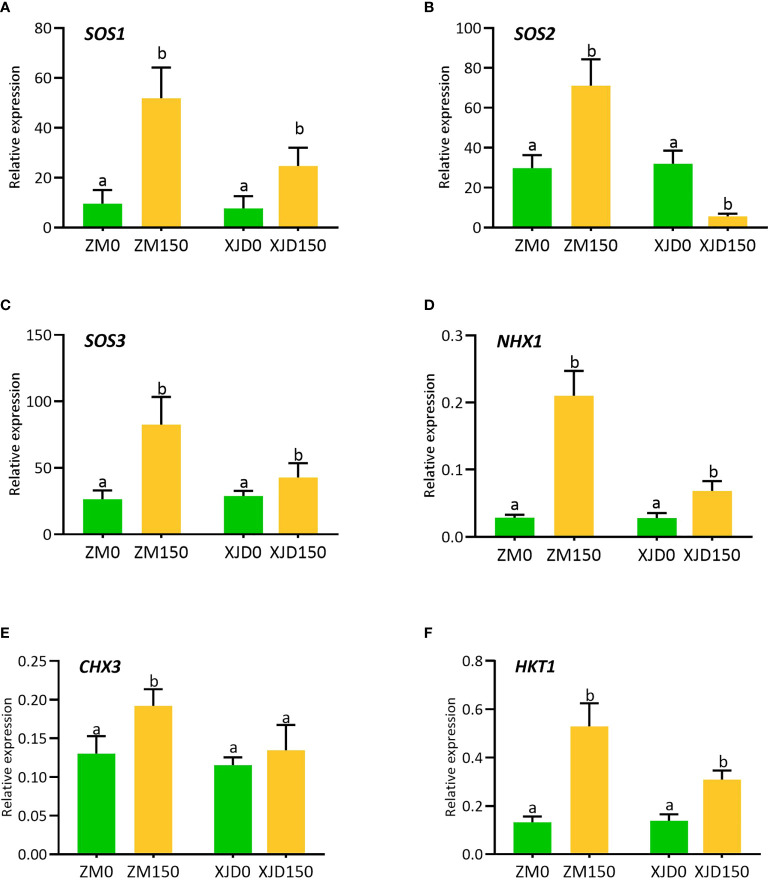
Transcript levels of Na^+^/H^+^ exchanger and Na^+^/K^+^ transporter genes involved in Na^+^ extrusion, K^+^ uptake, and translocation in two contrasting alfalfa under salt stress. Relative expression of *SOS1* **(A)**, *SOS2* **(B)**, *SOS3* **(C)**, *NHX1* **(D)**, *CHX3* **(E)**, and *HKT1* **(F)** in roots of Zhongmu (ZM) and Xingjiang Daye (XJD) under salt stress. *MsActin* gene was used to normalize the transcript abundance. The numeric zero (0) indicates salt-untreated (control) plant, while the numeric 150 indicates 150 mM salt treatment. Different letters above the column bar indicate significant differences among the means ± SD of treatments (n = 3). Data were analyzed by ANOVA and Tukey’s tests at *p* ≤ 0.05 significant level.

The expression of several ion transporter genes significantly responded to salt stress in alfalfa (**Figure 5**). The expression of *SOS1* was significantly induced in ZM roots than in that of XJD (**Figure 5A**). However, the *SOS2* and *SOS3* showed differential expressions in XJD. The expression of *SOS2* upregulated in ZM roots under salt stress, while it significantly declined in XJD (**Figure 5B**). The expression of *SOS3* was significantly higher than that of XJD (**Figure 5C**). Another ion exchanger *NHX1* was highly induced in ZM roots in response to salt stress, while it also was induced in XJD roots but the expression was lower than that of ZM (**Figure 5D**). The expression of *CHX3* was significantly induced by salt stress in ZM, while it showed no significant difference in XJD under salt and control conditions (**Figure 5E**). The expression of potassium importer *HKT1* was higher in ZM than in XJD in response to salt stress (**Figure 5F**).

### Responses of lignin biosynthetic genes under salt stress

**Figure 6:**
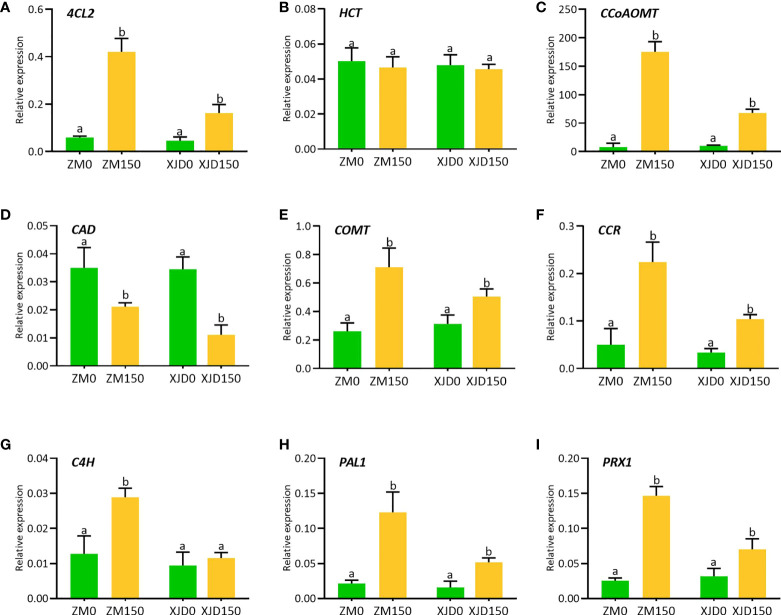
Transcript levels of various genes involved in lignin biosynthesis pathways as influenced by salt stress in two contrasting alfalfa. Relative expression of *4CL2* **(A)**, *HCT* **(B)**, *CCoAOMT* **(C)**, *CAD* **(D)**, *COMT* **(E)**, *CCR* **(F)**, *C4H* **(G)**, *PAL1* **(H)**, and *PRX1* **(I)** in roots of Zhongmu (ZM) and Xingjiang Daye (XJD) under salt stress. *MsActin* gene was used to normalize the transcript abundance. The numeric zero (0) indicates salt-untreated (control) plant, while the numeric 150 indicates 150 mM salt treatment. Different letters above the column bar indicate significant differences among the means ± SD of treatments (n = 3). Data were analyzed by ANOVA and Tukey’s tests at *p* ≤ 0.05 significant level.

**Figure 7:**
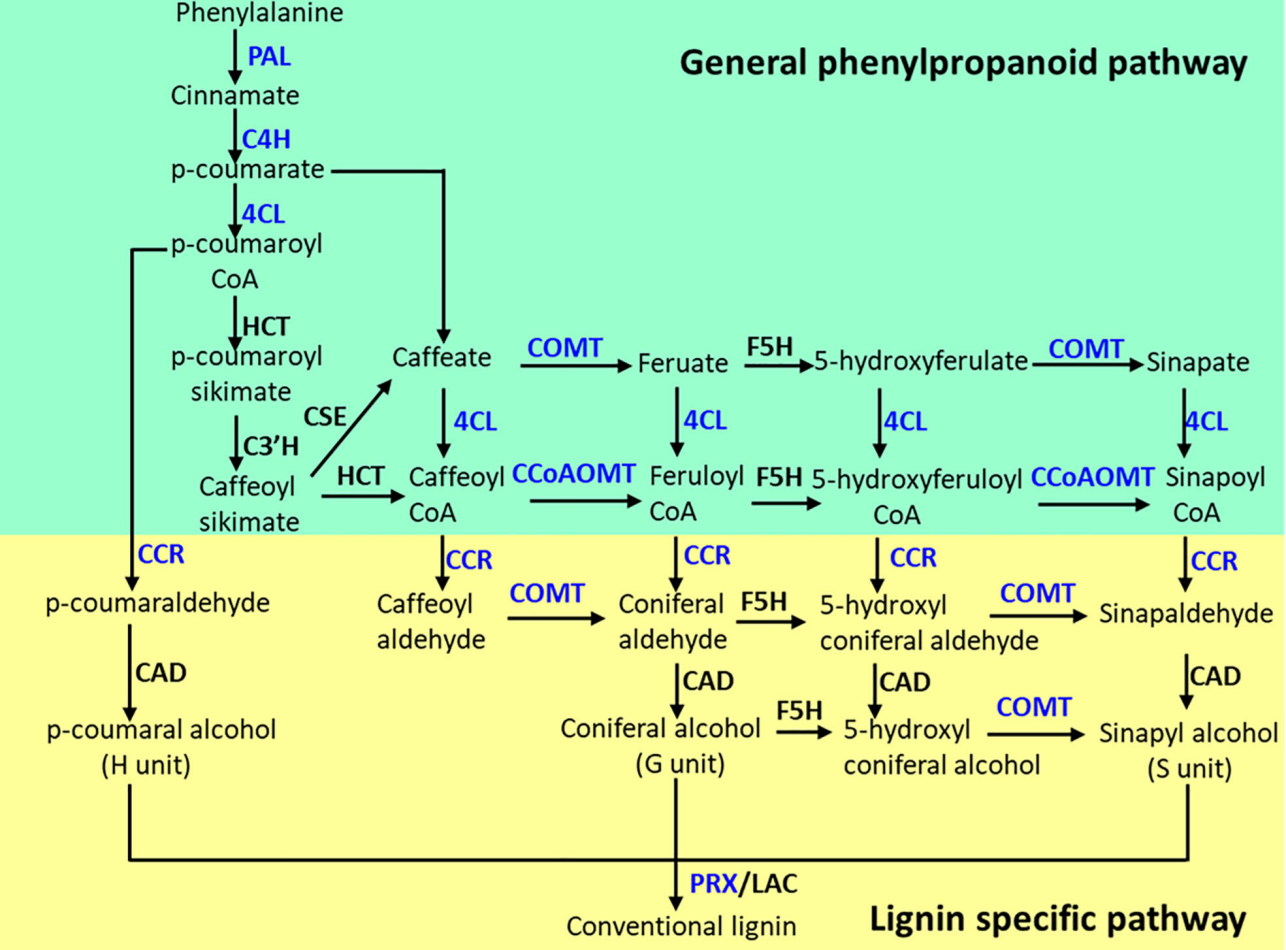
The phenylpropanoid (PP) and lignin-specific pathways as influenced by salt stress in alfalfa genotypes. Blue color indicates the genes were upregulated in Zhongmu (ZM) and/or Xingjiang Daye (XJD) under salt stress. The genes that did not show significant changes in expression are indicated in black color. PAL, phenylalanine ammonia-lyase; C4H, cinnamate 4-hydroxylase; 4CL, 4-coumarate CoA ligase; CHS, chalcone synthase; HCT, hydroxycinnamoyl CoA:shikimate/quinate hydroxycinnamoyl transferase; CCoAOMT, caffeoyl CoA 3-O-methyltransferase; F5H, ferulate 5-hydroxylase; COMT, caffeic acid O-methyl transferase; CCR, cinnamoyl CoA reductase; CAD, cinnamyl alcohol dehydrogenase; PRX, peroxidase.

The lignin biosynthesis–related genes showed differential expression patterns between the two contrasting alfalfa genotypes in response to salt (**Figure 6**). The expression of *4CL2* transcripts in roots of ZM was much higher than that of XJD (**Figure 6A**). The expression of *HCT* did not show any significant changes in two contrasting genotypes among the four treatments (**Figure 6B**). However, the *CCoAOMT* transcript showed a significant upregulation in ZM and XJD under salt stress compared with control plants, wherein the expression level was higher in ZM than in XJD (**Figure 6C**). The expression of *CAD* significantly declined in salt-treated plants of both genotypes compared with control plants, and the transcript response was much lower in XJD under salt (**Figure 6D**). Lignin biosynthesis related to some other genes prominently responded because of salt stress. The expression level of *COMT* was much higher in ZM than in XJD (**Figure 6E**). An almost similar pattern of expression was found in case of the *CCR* gene (**Figure 6F**). The expression of *C4H* was significantly upregulated in ZM under salt, while the *C4H* did not show any significant variation in XJD (**Figure 6G**). However, the expression level of *PAL1*was much higher in ZM than in XJD (**Figure 6H**). The last gene of lignin biosynthesis pathway *PRX*1 was largely upregulated in ZM compared with controls; it was also induced in XJD, but the expression was lower than that of ZM (**Figure 6I**). The transcript level of various genes involved in the phenylpropanoid (PP) and lignin-specific pathway influenced under salt stress in two contrasting alfalfa cultivars were represented in blue color (**Figure 7**).

### Changes in osmolytes and antioxidant enzymes in response to salt stress

**Figure 8:**
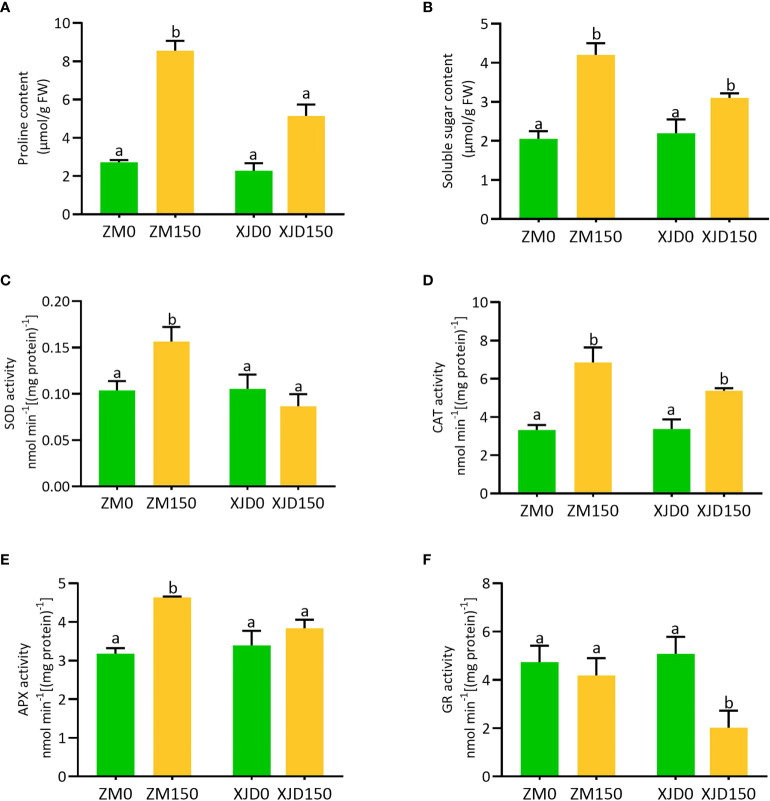
Regulation of osmolytes and antioxidant enzymes in two contrasting alfalfa under salt stress. Regulation of proline **(A)**, soluble sugar **(B)**, SOD **(C)**, CAT **(D)**, APX **(E)**, and GR **(F)** in Zhongmu (ZM) and Xingjiang Daye (XJD) under salt stress. The numeric zero (0) indicates salt-untreated (control) plant, while the numeric 150 indicates 150 mM salt treatment. Different letters above the column bar indicate significant differences among the means ± SD of treatments (n = 3). Data were analyzed by ANOVA and Tukey’s tests at *p* ≤ 0.05 significant level.

The accumulation of osmolytes and antioxidant enzymes were considerably altered under salt stress in two contrasting alfalfa genotypes (**Figure 8**). The concentration of proline was significantly induced in roots of both genotypes in response to salt as opposed to controls (**Figure 8A**). However, the level of accumulation was higher in ZM than in XJD. The concentration of soluble sugar showed almost a similar accumulation pattern to the proline (**Figure 8B**). We checked the response of antioxidant defense mechanisms of two contrasting alfalfa genotypes against the elevated ROS induced by salt stress. The activity of SOD significantly increased in response to salt stress in the root of ZM compared with untreated control plants, while this activity was reduced in XJD but no significant changes occurred because of salt stress (**Figure 8C**). The activities of CAT and APX were significantly induced in both genotypes compared with controls, whereas the activity was higher in ZM than in XJD under salt (**Figure 8D**). However, the APX activity was significantly induced in ZM relative to untreated plants but this enzyme showed no significant changes under salt stress in XJD (**Figure 8E**). The activity of GR significantly declined in XJD compared with control plants, while the activity of this enzyme did not show any significant alteration in ZM (**Figure 8F**).

## Discussion

This study explored a combination of differential mechanisms underlying salt stress tolerance in alfalfa. Plant response to salt stress provides novel features of major physiological and molecular traits, which associated with salt tolerance. However, salt tolerance efficiency can be useful to the plant breeder and the farmer for improving alfalfa production in salt-affected soils.

### Physiological variations in response to salt stress

Our study refers to tolerant ZM genotype with photosynthesis maintaining ability showed as a strategy to cope with excess salt accompanied by the well regulation of chlorophyll score in leaves. Moreover, control of excess Na^+^ absorption and its transport to aerial parts is one of the mechanisms that ZM possesses under salt stress. The reduction of photosynthetic efficiency in sensitive XJD indicates that salt stress severely affects the plant that causes photosynthesis damage. However, it is also possible that 150 mM salt could affect the alfalfa seedling, which causes photosynthesis disturbance (Li et al., 2010). In addition, salt-stressed plants possess higher susceptibility to PSII induced in a lower photosynthesis rate (Hichem et al., 2009). Furthermore, salt stress involving photo-oxidation in PSII may occur, damaging the photosynthetic apparatus (Stefanov et al., 2022). In this study, the remarkable changes of Fv/Fm ratio in XJD because of salt stress indicated the disturbance with photo inhibition of PSII, which significantly affected the plant growth attributes in alfalfa. Such adverse effect of salt on PSII was lower in the salt-tolerant ZM genotype. It was evidenced that salt stress causes photo inhibition, owing to induction of inactive reaction center, reduction of absorption flux, and low transfer of electrons per reaction center; these changes in PSII decline the maximum quantum yield of PSII (Fv/Fm) (Salim Akhter et al., 2021). Furthermore, salt-induced oxidative stress can decline PSII activity by damaging the reaction centers of PSII (Betzen et al., 2019). Therefore, photosynthesis impairment in XJD could be the outcome of oxidative damage.

The reduction of RWC% as a consequence of reduced osmotic potential might be associated with a concurrent increase in osmotic stress in XJD. As it was documented, the excess uptake of Na^+^ through root zone induces water and osmotic stress in plants (Arif et al., 2020). The reduction of root and shoot biomass, particularly salt-sensitive XJD, indicates that morphological inhibition is one of the adverse effects of salt stress. Reduction of plant biomass is an indication of the vulnerability of plant to salt stress (Buttar et al., 2021). However, the salt-tolerant ZM is comparably capable of coping with salt stress by adaptive mechanisms. Plants mitigate salt stress by conveying distinct mechanisms in roots and shoots (Zhao et al., 2020). In this study, salt-adaptive response in ZM ultimately provides better growth compared with XJD.

### Accumulation of Na^+^ and K^+^ ions and regulation of Na^+^/H^+^ exchangers and Na^+^/K^+^ transporter genes

Our study clearly shows that two alfalfa genotypes differ significantly in Na^+^ and K^+^ accumulation. One of the key findings was the ability of maintaining lower Na^+^ accumulation in the root and shoot than XJD and was efficient in retaining higher K^+^ accumulation, thus conferring ZM a higher K^+^/(K^+^ + Na^+^) ratio. Salt tolerance of a species often depends on the capability of maintaining optimum K^+^/(K^+^ + Na^+^) ratio by a strategy of decreasing Na^+^ and increasing K^+^ accumulation in cells (Munns and Tester, 2008). The halophytes showed the parallel feature of Na^+^ and K^+^ accumulation in cells (Wang et al., 2009). Salt-tolerant *Thellungiella salsugineea* is a close relative of *A. thaliana*, and their genome provides evidence about the nature of defense mechanisms with response to salt tolerance (Wu et al., 2012). A comparable difference of Na^+^ and K^+^ accumulation in these two species has been evidenced in terms of salt tolerance (Orsini et al., 2010). However, our present study clearly indicates that ZM is involved in higher tolerance to salt stress than XJD, as proven by declined Na^+^ content and elevated K^+^/(K^+^ + Na^+^) ratio in alfalfa under salt stress.

Furthermore, we studied the molecular responses underlying the salt tolerance in alfalfa. The greater expression *SOS1* was involved in mediating Na^+^ extrusion from the root cells, which leads to less accumulation of Na^+^ under salt stress. A correlation of the *SOS1* expression and salt tolerance has been documented in *Thellungiella* (Oh et al., 2009). Therefore, our current study suggests that *SOS1*-mediated Na^+^ extrusion is a key mechanism that maintains K^+^/(K^+^ + Na^+^) ratio in ZM, thus conferring salt tolerance in alfalfa.

Tissue-specific control of Na^+^ accumulation is one of the key approaches to reducing the toxicity of Na^+^ in plants under salt stress (Wu et al., 2015). The Na^+^/H^+^ antiporter *NHX1* is a transmembrane protein that plays a crucial role in Na^+^ homeostasis to enhance salt stress (Fan et al., 2015). Several studies revealed that the transcript of *NHX1* was markedly increased in root or shoot cells and found a positive correlation between the expression of *NHX1* and Na^+^ homeostasis under salt stress (Wu et al., 2015; Zhang et al., 2017). In our present study, the high expression of *NHX1* in ZM roots might be an indication of an Na^+^ homeostasis by limiting excessive Na^+^ into root cells, as a lower amount of Na^+^ content was obtained in ZM than in XJD. *HKTs* belong to a class of transmembrane proteins, which are involved in regulating Na^+^ and K^+^ transport in higher plants (Munns and Tester, 2008). In wheat, the *HKT1* expression in the root and leaf promoted K^+^ uptake (Schachtman and Schroeder, 1994). Furthermore, several studies documented that *HKTs* mediate Na^+^ retrieval from the xylem vessel of roots by preventing overaccumulation of Na^+^ (Munns and Tester, 2008; Zhang et al., 2017). In this study, the high expression of *HKT1* in ZM roots under salt stress indicates *HKT1* might be involved in unloading Na^+^ that was mediated by the K^+^ level, which helped to alleviate Na^+^ toxicity in ZM.

### Regulation of lignin biosynthesis genes under salt stress

The PP pathway is crucial for biosynthesis of monolignols; these monolignol units are the building blocks of lignin (Boerjan et al., 2003). In this PP pathway, the three key enzymes PAL, C4H, and 4CL catalyze the first three steps and serve as precursor for all the downstream metabolites. With these three enzymes, other PP biosynthetic enzymes that work downstream of 4CL including HCT, C3′H, CSE, CCoAOMT, COMT, CCR, F5H, and CAD are essential for normal lignin biosynthesis. In this study, we measured total lignin accumulation in roots, stems, and leaves of two contrasting alfalfa genotypes under salt stress. The higher level of lignin accumulation in ZM compared with XJD indicates the elevation of cell wall lignification and/or thickening in ZM than in XJD under salt stress, which provides better salt tolerance in ZM. Our results were supported by the study of lignin induction in stems and leaves of drought-tolerant transgenic alfalfa (Wen et al., 2021), while our current study found a significant variation in lignin content in roots and stems of two contrasting alfalfa. Therefore, it is clear that stress-induced lignin accumulation is obvious, but the level of accumulation can vary based on plant genotypes and its different parts. Beside lignin determination, we investigated the molecular mechanisms underlying lignin biosynthesis in two contrasting alfalfa genotypes. The upregulation of *4CL2, CCoAOMT, COMT, CCR, C4H*, *PAL1*, and *PRX1* in ZM related to XJD under salt stress indicates that ZM might be adapted to salt by regulating lignin biosynthesis genes under salt stress. A recent study clearly documented that the accumulation of lignin and the high expression of *Ms4CL,MsCCoAOMT MsCAD*, *MsPER9*, and *MsPER43* genes regulate cell wall lignification with drought stress tolerance in plants (Wen et al., 2021). A similar study has proven that, in *Arabidopsis*, the solid expression of *CCoAOMT1, 4CL1, 4CL2, COMT, PAL1, PAL2*, and *AtPrx52* genes play a critical role in salt adaptation (Chun et al., 2019). Furthermore, thickening of the cell wall under salt stress was also reported as a key factor that enhanced salt tolerance in plants (Liu et al., 2021). Under salt, there were alterations in the transcriptional level of candidate genes related to root lignification, cell wall solidification, and thickening of vascular tissues (Byrt et al., 2018). In bermudagrass, RNA-seq study reveals genotypic variations in salt tolerance, and the discovered candidate genes were also involved in changes to lignin formation, where peroxidases regulate ROS homeostasis (Hu et al., 2015). In this study, the potential responses of *4CL2, CCoAOMT, COMT, CCR, C4H*, *PAL1*, and *PRX1* suggest that these genes are vital for the regulation of lignin biosynthesis, which greatly enhanced salt tolerance in ZM. However, this study also provides a new perspective in anatomizing molecular mechanisms underlying lignin biosynthetic genes with salt tolerance in alfalfa and other legumes.

### Variation of osmotic and oxidative stress adaptation in contrasting genotypes under salt stress

Accumulation of osmolytes such as proline, soluble sugar, and glycine betaine are well-recognized metabolic outputs to osmotic stress promoted by salt, drought, or other abiotic stresses (Sami et al., 2016; Meena et al., 2019). Plants frequently accumulate free proline and soluble sugar to alleviate osmotic stress, which is involved in maintaining membrane integrity with optimal water potential (Quilambo, 2004). Sugar is also involved in the regulation of photosynthesis, stress-responsive genes, antioxidant system, and alleviation of abiotic stress (Sami et al., 2016; Raza et al., 2022a). However, the higher concentration of proline and soluble in this study provided more support to ZM for coping salt-induced osmotic stress than XJD. Furthermore, ZM showed quantitative changes of oxidative stress indicators ( ${\text{O}}_{2}^{•-}$ and H_2_O_2_), whereas XJD was severely affected by salt-induced toxicity. This observation suggests that 150 mM was above the typical level for XJD to cope with activating oxidative damage. Generation of ROS is an obvious outcome of multiple abiotic stresses in plant species; the optimum number of ROS maintains normal plant growth, but a large number of ROS is involved in cellular injury, lipid peroxidation, cellular damage, or abnormal plant growth (Huang et al., 2019). However, the efficiency of ROS controlling by enzymatic and non-enzymatic systems is recognized by tolerant genetic lines (Mittler, 2002). In this study, the lower ROS ( ${\text{O}}_{2}^{•-}$ and H_2_O_2_) accumulation in ZM might occur because of the higher accumulation of ROS-scavenging major enzymes (SOD, CAT, APX and GR) in ZM, which protected ZM plants from oxidative damages under salt stress. The SOD is considered a front-liner defense that catalyzes ${\text{O}}_{2}^{•-}$ to O_2_ and H_2_O_2_ (Huang et al., 2019; Kabir et al., 2021). The higher SOD, CAT, and APX activity in ZM may be associated with improved salt tolerance than that of XJD. Furthermore, there is a remarkable reduction of GR activity in XJD under salt stress. This response suggests that XJD was not able to maintain the cellular redox balance of GSH/GSSG under salt stress, whereas GR in ZM was capable of balancing the redox state as the GR level was maintained even under salt stress. However, the overall antioxidant enzyme activity in ZM validates its oxidative defense under salt stress.

## Conclusion

This work explores the mechanistic insights of salt stress tolerance in alfalfa legume. Differential morphophysiological and molecular responses between two genotypes of alfalfa indicate their sensitivity to salt stress. We demonstrated the strategies of Na^+^ and K^+^ uptake and/or translocation by which ZM accumulated less Na^+^; maintained high K^+^/(Na^+^ + K^+^) ratio; and had higher expression of *SOS1, NHX1*, and *HKT1* in roots under salt. However, these strategies were not fully active in XJD. Studies also imply the lignin accumulation along with its biosynthesis genes *4CL2, CCoAOMT, COMT, CCR, C4H*, *PAL1*, and *PRX1* in ZM accounted for greater salt tolerance. Our study further reveals that the accumulation of ROS was diminished by the antioxidant enzymes that facilitated better adaptation of ZM to salt stress. These results open up new insights of salt stress alleviation that will help to deliver the elite genetic material for alfalfa breeding programs.

## Data availability statement

The original contributions presented in the study are included in the article/**Supplementary Material**. Further inquiries can be directed to the corresponding author.

## Author contributions

MR, K-WL, and AK conceived the idea and conducted the research. K-WL and S-HL carried out the investigation. JW, HP, and AK analyzed the data. MR wrote the original draft. AR, AES, and AK helped in writing, review, and editing the original draft. All authors contributed to the article and approved the submitted version.

## Funding

This study was supported by the RDA Fellowship Program of National Institute of Animal Science and Cooperative Research Program for Agriculture Science and Technology Development (Project No. PJ01592501), Rural Development Administration, Republic of Korea.

## Conflict of interest

The authors declare that the research was conducted in the absence of any commercial or financial relationships that could be construed as a potential conflict of interest.

## Publisher’s note

All claims expressed in this article are solely those of the authors and do not necessarily represent those of their affiliated organizations, or those of the publisher, the editors and the reviewers. Any product that may be evaluated in this article, or claim that may be made by its manufacturer, is not guaranteed or endorsed by the publisher.
