# Supplementary material for: Regulation of Na+/H+ exchangers, Na+/K+ transporters, and lignin biosynthesis genes, along with lignin accumulation, sodium extrusion, and antioxidant defense, confers salt tolerance in alfalfa
Source: Front Plant Sci. 2022 Nov 7;13:1041764. doi: 10.3389/fpls.2022.1041764 (PMC9676661; doi:10.3389/fpls.2022.1041764)
Supplement: Supplementary file 1 [file Table_1.docx]

Supplementary Material

# Supplementary Table 1

A list of primers used for the gene expression analysis

| **Gene name** | **Forward primer** | **Reverse primer** | **Gene ID/Accession** |
| --- | --- | --- | --- |
| *Actin* | TTCTCACCACACTTCTCGCC | CCAGCCTTCACCATTCCAGT | JQ028730 |
| *SOS1* | CAGGGATATTGCATGCTGCG | TGGCCAGGGATGTCTACCAT | XM_039828782 |
| *SOS2* | GGTGTTTACCACCGTGACCT | ACCATCGTAACCCTGTCCAC | AY099621 |
| *SOS3* | AGGTGTCTTCCATCCAAGCG | GCACGAAAGCCTTATCCACC | NM_001203448 |
| *NHX1* | ACTTCTTGGAAGAGCAGCGT | AGTTGAGTATGCCCCGACAC | GU265772 |
| *CHX3* | CATTCCTCCGATCTTGCGGT | GATTCCCGGGACGGACAAAT | NM_122196 |
| *HKT1* | ACGTAGGGTTGTCAACAGGA | TGTTAGCTAGGACAGGTGCC | XM_024785691 |
| *4CL2* | TCTGATGCTGCTGTCGTACC | CCTGAGGCGGCTTTAGGAAT | XM_013603074 |
| *HCT* | AGCAAGGTGCTTGTCCCATT | CGCGAATAATCAACGGCTGG | KR611925.1 |
| *CCoAOMT* | AGGTTGGGGGATTAATCGGT | TGCGGCGACATAGGGTAATC | XM_013601988 |
| *CAD* | GGCATGAAGTGGTTGGTGAG | AGTGATCTTGCCGTCAGTGT | XM_013614607 |
| *COMT* | TGAGCATGTTGGTGGAGACAT | ACATGTTGACAATGACACCCG | JN850020 |
| *CCR* | GAGAATCACGAGGCAGACGG | GGCCTGAGTTTCCAACCCAA | XM_039833912 |
| *C4H* | CAAACTCCCACCAGGTCCAA | GTGGAGGACTTCTTTGGCGA | HM627322 |
| *PAL1* | TCGGTCAACGACAACCCTTT | TGCAGTGAGATTCGAAGGCA | JN849822 |
| *PRX1* | CCGAATGGGTGCCTCTATCC | GGGGCATACGTTCTCTACGG | EF456703 |
